# Natural conception complicated by spontaneous ovarian hyperstimulation syndrome in the setting of severe primary hypothyroidism: A case series

**DOI:** 10.1097/MD.0000000000044825

**Published:** 2025-10-10

**Authors:** Wenming Zhuang, Lian Wu, Ruili Shao, Kaiheng Zhang, Lingfang Ye

**Affiliations:** a Department of Obstetrices and Gynecology, Women and Children’s Hospital Affiliated to Ningbo University, Ningbo, Zhejiang Province, China; b Department of Obstetrices and Gynecology, Haishu Women & Children’s Hospital, Ningbo, Zhejiang Province, China.

**Keywords:** case report, hypothyroidism, natural conception, ovarian cystic mass, ovarian hyperstimulation syndrome

## Abstract

**Rationale:**

Spontaneous ovarian hyperstimulation syndrome (sOHSS) occurring in naturally conceived pregnancies is exceedingly rare, particularly when associated with untreated primary hypothyroidism. Misdiagnosis may result in unnecessary interventions, whereas delayed diagnosis can worsen disease progression.

**Patient concerns:**

Four pregnant women presented with bilateral, multiloculated ovarian cysts (>10 cm), markedly elevated thyroid-stimulating hormone levels, and mild-to-moderate dilutional anemia. Two patients exhibited significant abdominal distension accompanied by markedly elevated β-human chorionic gonadotropin levels, while 3 displayed classic hypothyroid symptoms.

**Diagnoses:**

All cases were diagnosed with severe primary hypothyroidism complicated by secondary sOHSS.

**Interventions:**

Three patients were treated with levothyroxine (LT4). The fourth patient underwent an unnecessary laparoscopic cystectomy due to initial misdiagnosis; postoperatively, fetal cleft lip and palate were identified on ultrasound.

**Outcomes:**

The 3 patients receiving LT4 therapy achieved complete resolution of their ovarian cysts. The misdiagnosed case resulted in pregnancy termination following the detection of fetal abnormalities.

**Lessons:**

In the evaluation of bilateral ovarian cysts during naturally conceived pregnancy, sOHSS secondary to hypothyroidism should be considered. Multidisciplinary consultation is advised, along with a comprehensive assessment of thyroid function, β-human chorionic gonadotropin, and follicle-stimulating hormone receptor levels. Timely medical intervention with levothyroxine can prevent disease progression and promote cyst resolution, thereby avoiding unnecessary surgical procedures. Enhanced clinical recognition of this rare yet treatable condition is critical for ensuring optimal patient management.

## 1. Introduction

Ovarian hyperstimulation syndrome (OHSS) is typically a form of hyperreactio luteinalis^[1]^ Mild cases may be asymptomatic or present with nausea, vomiting, and abdominal distension. Moderate to severe OHSS occurs in 3 to 8% of in vitro fertilization cycles.^[2]^ Spontaneous ovarian hyperstimulation syndrome (sOHSS) is exceedingly rare, with reported incidence rates ranging from 0.2 to 1.2%.^[3]^ Previous reports of sOHSS have primarily involved non-pregnant women, often in association with conditions such as polycystic ovary syndrome, gonadotropin-secreting pituitary adenomas, pituitary enlargement, and hypothyroidism.

Hypothyroidism, one of the most common endocrine disorders among women of reproductive age,^[4]^ has been increasingly recognized as a potential trigger for sOHSS. Severe ovarian hyperstimulation syndrome further exacerbates pregnancy-related risks, including miscarriage, stillbirth, preterm delivery, and low birth weight.^[5]^ Moreover, uncontrolled hypothyroidism increases the likelihood of maternal complications such as anemia, gestational hypertension, placental dysfunction, placental abruption, fetal growth restriction, and teratogenic effects. However, the literature on natural pregnancy-associated sOHSS with concurrent hypothyroidism remains limited to isolated case reports,^[6–15]^ and there is no clear clinical guidance regarding optimal management and diagnosis strategies. In this report, we present 4 cases of ovarian hyperstimulation induced by hypothyroidism during natural pregnancy.

Each of these 4 cases exhibits distinct features. In Case 1, a large ovarian cyst detected during pregnancy was not initially suspected to be related to sOHSS. Consequently, HCG was never measured and thyroid function was not evaluated during prenatal visits until the patient was hospitalized, at which point levothyroxine (LT4) therapy was initiated. In Case 2, the diagnosis was established early in pregnancy, and LT4 treatment was administered. However, the couple opted for medical termination due to concerns regarding potential adverse effects of severe hypothyroidism on the fetus. Case 3 involved a timely diagnosis and initiation of LT4 therapy during the second trimester, leading to an uneventful pregnancy outcome. In Case 4, the patient had a known history of hypothyroidism. Nevertheless, the potential contribution of poorly controlled thyroid dysfunction was overlooked, and the patient underwent laparoscopic ovarian cystectomy.

This case series underscores the importance of excluding hypothyroidism in pregnant women presenting with bilateral adnexal masses. Early detection of thyroid dysfunction and prompt initiation of LT4 therapy can help avoid unnecessary surgical interventions. These findings highlight the need for greater clinical awareness of this underrecognized condition and provide practical insights for reproductive endocrinologists, obstetricians, and surgeons.

## 2. Case report

### 2.1. Study subjects

The study included pregnant women between 21 and 30 years of age, with a height ranging from 1.55 to 1.60 meters, and educational levels spanning from junior high school to college. All participants had a history of menarche at 13 to 15 years of age and regular menstrual cycles, and conceived naturally. In Case 4, the patient had a known history of hypothyroidism and was treated with LT4, but the condition was poorly controlled. Past medical history, family history, and psychosocial history are unremarkable in the other cases. Three patients presented with typical hypothyroid facies, and 2 exhibited abdominal distension. Further details are provided in Table 1.

**Table 1:** Clinical features and managements of 4 cases colored in yellow.

Item	Case 1	Case 2	Case 3	Case 4
Age	30-yr-old woman primipara	21-yr-old primigravida	27-yr-old primigravida	25-yr-old woman multipara
TPAL	0-0-2-0	0-0-0-0	0-0-0-0	1-0-1-1
Gestation week	39 wk of gestation	9 2/7 wk of gestation	13 2/7 wk of gestation	17 6/7 wk of gestation
Main complaint	Bilateral ovarian cystic masses persisting at 18 wk of gestation	A 7-d history of abdominal distension	An 8-d history of abdominal distension	Bilateral ovarian masses identified at 13 wk of gestation
Physical examination	Physical examination upon admission revealed a dull appearance, sluggishness, slow speech, and a bloated face. The abdomen was protuberant, and palpation revealed an enlarged mass without tenderness. Gynecological examination showed normal female external genitalia with normal pubic hair distribution.	The patient appeared pale, with a bloated face and slow speech. Breast examination revealed no masses or discharge. Mild tenderness and distension were noted in both lower abdominal quadrants. Gynecological examination showed normal external genitalia with typical pubic hair distribution.	Physical examination revealed an anemic appearance, bilaterally enlarged cystic masses, and a gravid uterus consistent with 3 mo of gestation.	Physical examination revealed a lethargic patient with a bloated face and slow speech. The abdomen was protuberant but non-tender, with a well-healed surgical scar in the lower midline. Axillary and pubic hair distribution was normal.
Imaging report	Bilateral multiloculated ovarian cysts, measuring 13.4 × 12.2 cm (right ovary) and 9.0 × 6.2 cm (left ovary), were initially detected by abdominal ultrasound at 18 wk of gestation. Magnetic resonance imaging revealed enlargement of the right ovarian cyst to 200 mm during hospitalization (Fig. 1A).	Abdominal ultrasound revealed bilateral multiloculated ovarian cysts, measuring 12.1 × 8.7 cm (left ovary) and 11.3 × 10.7 cm (right ovary), along with bilateral pleural effusions (right: 7.1 cm, left: 4.8 cm) (Fig. 2).	Ultrasound revealed bilateral multiloculated ovarian cysts, measuring 16 × 12.4 cm (left ovary) and 12.1 × 12.1 cm (right ovary) (Fig. 3).	Pelvic ultrasound revealed bilateral multiloculated ovarian cysts, which had increased in size to 12 cm (left ovary) and 14 cm (right ovary) (Fig. 4A).
Treatment	The patient was initially treated with levothyroxine 100 μg daily.	The patient was started on levothyroxine 100 μg daily.	The patient was treated with levothyroxine 100 μg daily.	The dosage of levothyroxine (LT4) was adjusted from 100–150 μg once daily.
Follow-up and pregnancy outcome	On the third day of admission, a cesarean section was performed due to prolonged second-stage labor and fetal anterior asynclitism. During the procedure, 300 mL of clear ascites was observed, and both ovaries were multicystic, with the right ovarian cyst extending below the costal margin. Aspiration of superficial cysts yielded 2.0 L of serous fluid. Thinned ovarian tissue without cyst wall was noted upon incision of one cyst. Intraoperative histologic examination of frozen sections confirmed benign cysts, with the final histologic diagnosis being luteinized follicular cysts (Fig. 1B). Levothyroxine therapy was continued, with thyroid function monitored every 2–4 wk postpartum until stabilization. The dosage was subsequently adjusted to a maintenance dose of levothyroxine 50 μg once daily. Two months postpartum, follow-up sonography showed normal ovarian size, and thyroid-stimulating hormone levels had normalized.	Due to concerns about potential adverse effects on fetal neurological development, the parents opted for pregnancy termination after a week of inpatient care. Post-discharge, the patient was monitored regularly every 2–4 wk, and levothyroxine therapy was maintained at 100 µg/day. Three months later, her TSH levels normalized, and pelvic ultrasonography showed resolution of the ovarian cysts to normal size.	Her symptoms improved significantly within 5 d, and she was discharged. Follow-up evaluations were conducted every 2–4 wk, and the levothyroxine dose was gradually increased to 75 µg/day. Post-discharge, she achieved euthyroidism within 1.5 mo. Serial ultrasounds showed no significant ovarian enlargement 3 mo later. At 39 wk of gestation, she underwent a cesarean section for macrosomia. Intraoperative findings revealed a normal-sized left ovary and a slightly enlarged right ovary with features consistent with polycystic ovary disease.	Laparoscopic cystectomy was performed for diagnostic and therapeutic purposes on the second day of admission. Intraoperative findings confirmed bilateral multiloculated ovarian cysts with smooth surfaces and 200 mL of clear ascites. Histopathological examination revealed ovarian luteinized follicular cysts (Fig. 4B). Four days postoperatively, fetal cleft lip and palate were detected on ultrasound, leading to elective termination of pregnancy 8 d after surgery. The patient was monitored regularly every 2–4 wk, then achieved euthyroidism 43 d post-abortion.
Subsequent pregnancy	The patient achieved another spontaneous conception 2 yr later and remained euthyroid throughout the pregnancy, delivering a term male infant by cesarean section.	Eleven months later, the patient achieved spontaneous conception and remained euthyroid throughout the pregnancy. She delivered a term male infant via cesarean section.	No current plans for reproduction.	The patient conceived again 2 mo post-abortion and was euthyroid on LT4 125 μg daily. noninvasive prenatal testing (NIPT) results were normal. However, at 24 3/7 wk of gestation, fetal total anomalous pulmonary venous connection, a small left atrium and ventricle, and an atrial septal defect were diagnosed via ultrasound, resulting in another pregnancy termination.
Current medication	Levothyroxine replacement therapy was maintained at 50 µg daily.	Levothyroxine therapy was maintained at 100 µg daily.	Levothyroxine therapy was maintained at 75 µg daily.	Levothyroxine therapy was maintained at 125 µg daily.

### 2.2. Laboratory findings

Decreased levels of free triiodothyronine (FT3) and free thyroxine (FT4) were observed to varying degrees, while thyroid-stimulating hormone (TSH) was significantly elevated. Prolactin (PRL) levels were also elevated in all cases. Moderately increased levels of CA125 and alpha-fetoprotein (AFP) were detected. Hemoglobin (Hb) and hematocrit (HCT) levels were below normal ranges, and human chorionic gonadotropin (HCG) was markedly elevated in 3 cases. (In Case 1, serum HCG was not tested since sOHSS was not clinically suspected.) Detailed laboratory results are summarized in Table 2.

**Table 2:** Laboratory tests for 4 cases.

Item	Case 1	Case 2	Case 3	Case 4	Reference range
TSH	>100	>100	>100	105.95	0.47–5 mIU/L
FT4	0.0	0.0	0.09	0.37	0.58–1.64 ng/dL
FT3	1.4	2.0	1.4	2.1	2.3–4.2 pg/mL
PRL	50.13	47.35	74.9	53.68	5–25 ng/mL
AFP	178.4	10.09	15.92	89.3	0-9 ng/mL
CA125	51.10	429.2	290.2	36.20	0–35 U/mL
HCG	Not tested	>200,000	267,594	75,002	<7 mIU/mL
HCT	27.5	25.8	24.9	29.4	35–45%
HB	8.6	9.0	7.4	9.5	11–15 g/dL

AFP = alpha-fetoprotein, CA125 = carbohydrate antigen 125, FSH = follicle-stimulating hormone, FT3 =free triiodothyronine, FT4 =free thyroxine, HB = haemoglobin, HCG = human chorionic gonadotrophin, HCT = hematocrit, LH = luteinizing hormone, PRL =prolactin, TSH = thyroid-stimulating hormone.

### 2.3. Imaging findings

**Figure 1.**
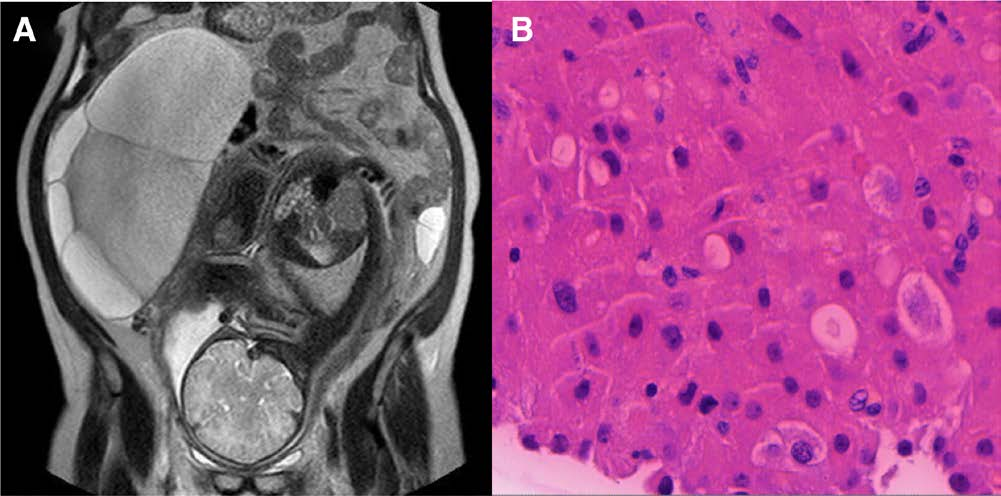
A 30-yr-old woman, gravida 3, para 0, who conceived naturally, was admitted at 39 wk gestation due to bilateral ovarian cystic masses persisting for over 4 mo. (A) Magnetic resonance imaging (MRI) scan demonstrated an abnormally enlarged right ovary with multiple cystic masses. (B) Histopathological analysis confirmed the presence of a multiloculated ovarian cyst, identified as a luteinized follicular cyst (magnification ×400).

**Figure 2.**
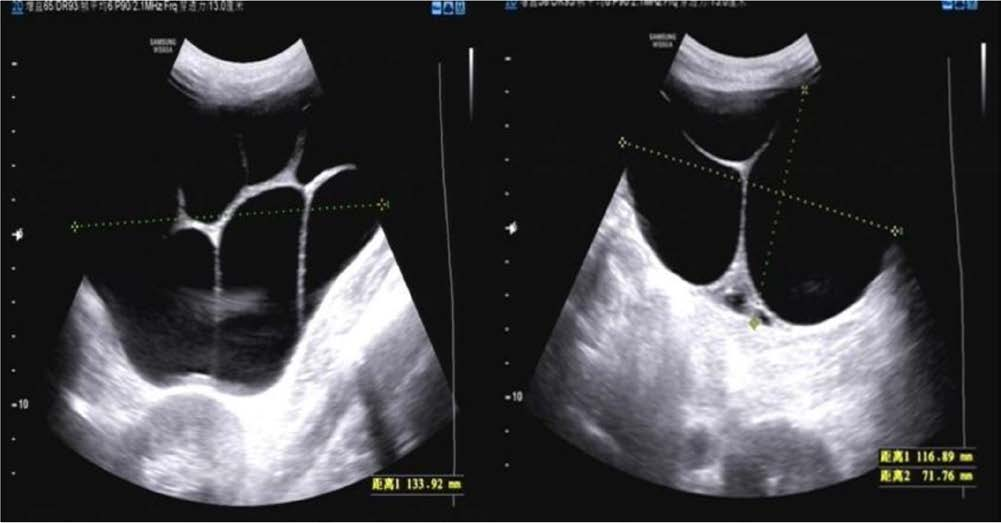
A 21-yr-old primigravida at 9 2/7 wk of gestation, presented with a 7-d history of abdominal distension. Ultrasonographic imaging revealed bilateral ovarian enlargement with multiple contiguous thin-walled cysts containing anechoic fluid.

**Figure 3.**
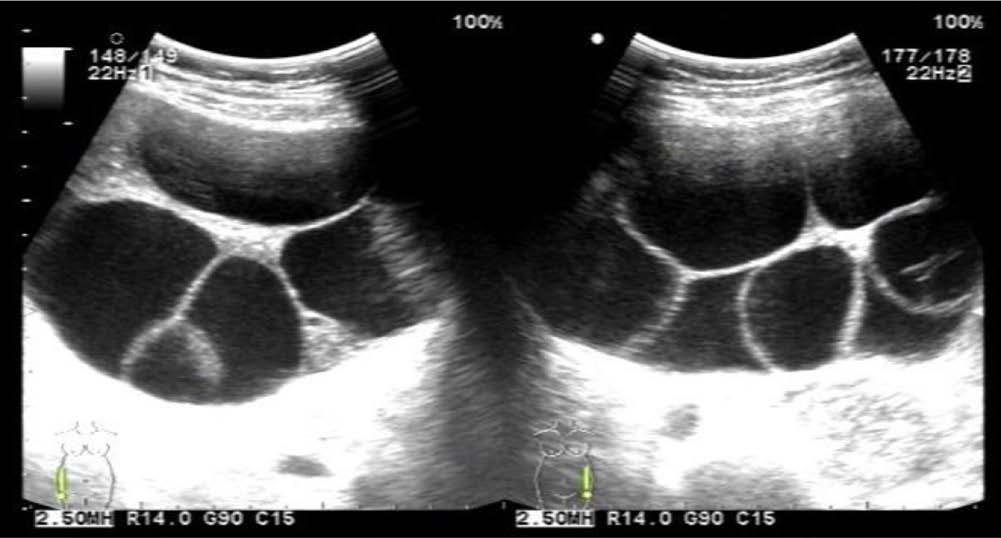
A 27-yr-old primigravida at 13 2/7 wk of gestation presented with an 8-d history of abdominal distension. Ultrasound findings indicated bilateral multiloculated ovarian cystic masses accompanied by spontaneous ovarian hyperstimulation.

**Figure 4.**
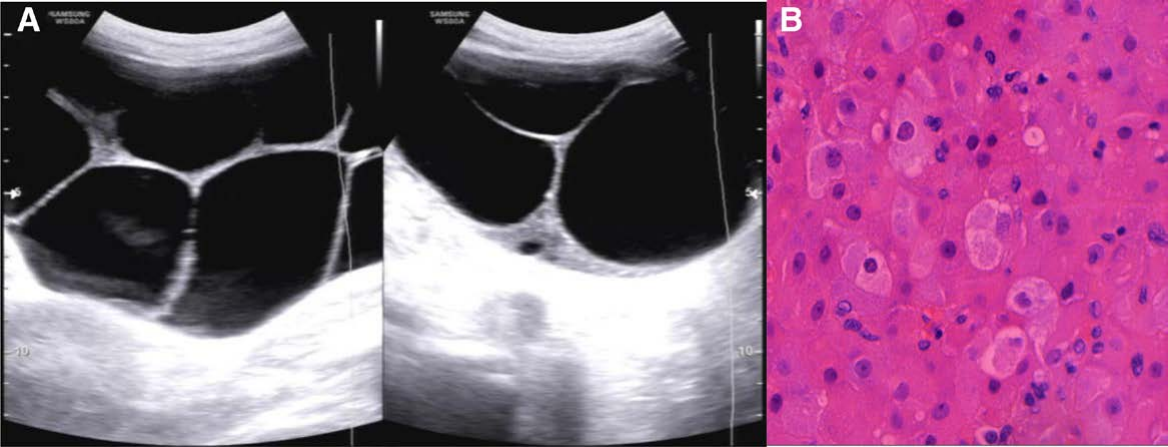
A 25-yr-old multipara presented at 17 6/7 wk of gestation with bilateral ovarian masses identified one month prior. (A) Pelvic sonography identified bilateral multiloculated ovarian cysts in the absence of abdominal pain or distention. (B) Pathological examination of the specimens from this case confirmed the diagnosis of ovarian luteinized follicular cyst (magnification ×400).

Ultrasonography revealed large multilocular cystic masses in the bilateral adnexal regions of all patients (Figs. 2, 3, and 4A). One patient underwent abdominal magnetic resonance imaging (MRI), which confirmed massive bilateral abdominal masses, with the right-sided lesion measuring up to 22 cm in maximum diameter (Fig. 1A). Additional imaging characteristics are provided in Table 1.

### 2.4. Pathological findings

Pathological examination confirmed that the ovarian cysts in Cases 1 and 4 were luteinized follicular cysts (Figs. 1B and 4B). In Cases 2 and 3, the cysts resolved following LT4 supplementation, and no pathological specimens were obtained for evaluation.

### 2.5. Differential diagnosis

All 4 patients were diagnosed via ultrasound with bilateral giant ovarian cysts during pregnancy. Laboratory findings revealed markedly elevated TSH levels (>100 mIU/L), hyperprolactinemia, moderate dilutional anemia, high HCG levels, as well as elevated CA125 and AFP. These abnormalities raised suspicion of either pregnancy-associated ovarian malignancy or Van Wyk–Grumbach syndrome (VWG).

Ovarian malignancies typically present on ultrasound as complex cystic structures with irregular solid components, heterogeneous echogenicity, irregular contours, surface nodularity, and detectable vascular signals. Although CA125 and AFP were elevated in all patients, it is important to note that during pregnancy, CA125 is largely secreted by decidual and amniotic tissues, while AFP is primarily produced by the fetal yolk sac and liver, with physiological fluctuations across gestational stages. Current clinical guidelines caution against immediately attributing elevated CA125 or AFP levels to ovarian malignancy during pregnancy. Instead, a comprehensive assessment incorporating imaging – such as ultrasound and MRI – along with serial measurements of tumor markers, is recommended. In these 4 cases, ovarian enlargement was predominantly bilateral and multicystic, characterized by anechoic fluid-filled cavities, making malignancy less likely. Following thyroid hormone replacement therapy, the ovarian cysts regressed completely in all patients. Cases 1 and 4 were pathologically confirmed as ovarian luteinized cysts.

VWG syndrome is a distinct manifestation of long-standing primary hypothyroidism, most frequently reported in prepubertal children, with occasional cases in adolescents and rare instances in adults. Prepubertal presentations typically include primary hypothyroidism, unilateral or bilateral ovarian cysts, precocious puberty, delayed bone age, and galactorrhea, often in the absence of axillary or pubic hair development.^[16]^ Since all 4 patients conceived naturally and had no medical or menstrual history suggestive of precocious puberty before adolescence, along with normal axillary and pubic hair development, VWG syndrome was ruled out.

## 3. Discussion

The patients in Cases 2 and 3 exhibited favorable treatment responses, achieving complete resolution of symptoms following LT4 therapy. In contrast, Cases 1 and 4 demonstrated divergent clinical outcomes. In Case 1, a pregnant woman was found to have bilateral giant ovarian cysts during a second-trimester ultrasound examination. However, the outpatient physician failed to perform serum HCG testing and thyroid function, resulting in a missed diagnosis. Severe hypothyroidism was not detected until admission at 39 weeks of gestation, by which time the right ovarian cyst had enlarged to a maximum diameter of 22 cm. The demand for thyroid hormones increases during pregnancy, necessitating timely LT4 dose adjustments in women being treated for hypothyroidism. In Case 4, suboptimal LT4 dosing led to severe ovarian hyperstimulation syndrome (sOHSS). Clinical evaluation focused on ovarian cyst dimensions without investigating the underlying etiology, resulting in a missed diagnosis of hypothyroidism-induced bilateral giant ovarian cysts. Importantly, the association between hypothyroidism and cystic enlargement was overlooked, leading to the omission of multidisciplinary consultation and unnecessary surgical intervention. This iatrogenic cascade underscores the importance of multidisciplinary consultation in complex pregnancies, particularly when hypothyroidism may be a contributing factor.

sOHSS is defined as ovarian hyperstimulation occurring in the absence of pharmacological ovulation induction. Patients may present abdominal distension, abdominal pain, nausea, vomiting, rapid weight gain, and dyspnea. Preconception ultrasound or ultrasound during the conception cycle showed normal bilateral ovarian size, whereas post-conception ultrasound revealed bilateral polycystic ovarian enlargement or ascites. Laboratory findings may include hemoconcentration, leukocytosis, hypoalbuminemia, and renal dysfunction, among others. The etiology of sOHSS remains incompletely understood. Potential triggers include multiple pregnancies, gestational trophoblastic disease, polycystic ovary syndrome (PCOS), primary hypothyroidism, TSH or gonadotropin-secreting adenomas, and mutations in the follicle-stimulating hormone receptor (FSHR) gene.^[13–15,17–19]^ The pathophysiology of sOHSS involves the release of numerous vasoactive mediators from overstimulated ovaries, including vascular endothelial growth factor and pro-inflammatory cytokines (e.g., interleukins and tumor necrosis factor). These factors act synergistically to increase capillary permeability and activate the renin-angiotensin system.^[20]^ Consequently, intravascular fluid and proteins extravasate, leading to hemodynamic disturbances such as hypotension, tachycardia, and reduced renal perfusion (manifesting as hyperkalemia, hyponatremia, and decreased creatinine clearance). Hemoconcentration also occurs (evidenced by elevated hematocrit and leukocytosis), along with third-space fluid accumulation and intravascular dehydration.^[21]^ The pathogenesis may involve aberrant sensitivity of mutated FSHR to HCG^[22]^ Such mutations can lead to loss of ligand binding specificity and heightened responsiveness to HCG, particularly during the first trimester when HCG levels are elevated. Alternatively, hypersecretion of glycoprotein hormones (HCG, TSH, FSH, LH) with shared subunits may overcome receptor specificity. Elevated TSH can dose-dependently stimulate FSH and LH receptors, producing effects analogous to those of FSH and LH,^[1]^ resulting in excessive stimulation of wild-type FSHR by HCG or TSH. Another hypothesis proposes that variant forms of HCG or TSH in an autocrine environment may activate FSHR with heightened sensitivity.^[23]^ In this case series, FSHR gene analysis was not performed, as the clinical team did not pursue a definitive etiological diagnosis. Patients with OHSS produce estriol via the 16-hydroxylation pathway, which is less effective than estradiol in the gonadotropin feedback loop, leading to increased FSH release and ovarian hyperstimulation.^[24]^ All 4 patients exhibited dilutional anemia rather than hemoconcentration. Sridev et al suggest that while vasoactive substances released in iatrogenic OHSS primarily drive increased vascular permeability, sOHSS associated with elevated HCG, TSH, or FSHR mutations may not involve systemic extravascular efflux, resulting in hemodilution.^[13]^ Anemia in hypothyroidism may also arise from impaired erythropoiesis due to thyroid hormone deficiency, reduced erythropoietin production, and malabsorption of iron and vitamin B12 secondary to gastric acid deficiency.^[25]^ Untreated hypothyroidism or antibody-positive status increases the risk of anemia,^[26,27]^ while anemia itself is associated with an elevated risk of hypothyroidism in pregnancy.^[28,29]^

Maternal hypothyroidism during the first trimester is associated with adverse pregnancy outcomes, including congenital anomalies and impaired neurocognitive development in offspring. Specifically, maternal hypothyroidism has been linked to an increased risk of congenital heart defects in the fetus.^[30]^ Additionally, mutations in thyroid transcription factors, such as FOXE1-observed in patients with hypothyroidism or thyroid cancer-have been implicated in the pathogenesis of cleft lip and palate.^[31]^ The patient in Case 4 had a 3-year history of poorly controlled hypothyroidism and experienced 2 consecutive pregnancy losses: the first in 2020 due to fetal cleft lip and palate, and the second in 2021 due to a cardiac malformation. The parents declined genetic testing; therefore, chromosomal analysis was not performed for the malformed fetuses. This case highlights the critical importance of meticulous fetal anomaly screening in pregnant women with severe hypothyroidism, including comprehensive ultrasound evaluation for congenital heart disease, cleft lip and palate, choanal atresia, and other structural abnormalities.

A systematic review of PubMed using the keywords “hypothyroidism,” “sOHSS,” “natural pregnancy,” and “ovarian cyst” identified ten relevant cases reported between 1998 and 2025^[6–15]^ Diagnosis occurred between 6 and 22 weeks of gestation, and patient ages ranged from 22 to 34 years. All patients with abnormal thyroid function and elevated TSH levels received LT4 replacement therapy. Ovarian cysts resolved spontaneously within a period ranging from 2 weeks to 15 months. Genetic testing for FSHR gene mutations was performed in 2 cases. Delivery was achieved in 8 cases, 3 of which were by cesarean section; one of these involved aspiration of a large superficial ovarian cyst and wedge biopsy. Cardoso et al^[6]^ reported a case of spontaneous preterm labor, and Edward-Silva et al^[9]^ reported a preterm cesarean delivery. Two patients opted for termination of pregnancy.^[10,14]^ A comprehensive summary of these data is provided in Table 3.

**Table 3:** Clinical assessment of sOHSS with hypothyroidism in natural pregnancy.

References	Age	Gestation weeks	Abdominal pain/distension	Hemoconcentration	Anemia	FSHR mutation	LT4	Ovarian cyst progress	Pregnancy outcome
Nappi et al^[6]^	34	12	Yes	N/A	N/A	N/A	Yes	Significant reduction of ovarian size after 2 wk of medication	Spontaneous vaginal delivery of at 38 wk of gestation
Cardoso et al^[7]^	25	11–12	Yes	N/A	N/A	N/A	Yes	Total regression of the ovarian cysts at 24 wk	Vaginal delivery of a 1120 g male infant at 28 weeks
Borna et al^[8]^	30	20	Yes	No	No	N/A	Yes	Aspiration of a large superficial ovarian cyst and wedge biopsy were done. Sonography showed normal ovarian size 10 wk after delivery.	Cesarean section due to breech presentation at 38 weeks of gestation
Edwards-silva et al^[9]^	30	10 1/7	Yes	No	Yes	N/A	Yes	Total regression of the ovarian cysts at 22 wk of gestation	Cesarean delivery secondary to nonreassuring fetal heart tracing at 34 3/7 wk of gestation
Lussiana et al^[10]^	29	22	Yes	Yes	No	Yes	Yes	Total regression of the ovarian cysts 2 mo after abortion	Spontaneous abortion
Dieterich et al^[11]^	26	6	Yes	No	Yes	Yes	Yes	N/A	Vaginal delivery at 39 wk of gestation
Sridev et al^[12]^	22	9	Yes	No	Yes	N/A	Yes	Total regression of the ovarian cysts at 20 wk	Spontaneous vaginal delivery at 39 wk of gestation
Oliveira et al^[13]^	32	13	No	No	Yes	N/A	Yes	Complete regression of the cysts 8 mo after postpartum	Cesarean delivery due to preeclampsia at 37 wk of gestation
Guerra et al^[14]^	22	9	Yes	Yes	No	N/A	Yes	Total regression of the ovarian cysts 3 mo after artificial abortion	Voluntarily terminate the pregnancy
Alzebidi et al^[15]^	27	10	Yes	No	No	N/A	Yes	Complete resolution of ovarian cysts by 3 mo postpartum	Spontaneous vaginal delivery at term gestation
This case series									
Case 1	30	39	No	No	Yes	No	Yes	Aspiration of a large superficial ovarian cyst and wedge biopsy were done. Two months postpartum, follow-up sonography showed normal ovarian size.	Cesarean section due to prolonged second-stage labor and fetal anterior asynclitism
Case 2	21	9 2/7	Yes	No	Yes	No	Yes	Resolution of the ovarian cysts to normal size 3 mo later	Voluntarily terminate the pregnancy
Case 3	27	13 2/7	Yes	No	Yes	No	Yes	Complete regression of the cysts 3 mo later	Cesarean section for macrosomia at 39 wk of gestation
Case 4	25	17 6/7	No	No	Yes	No	Yes	Laparoscopic cystectomy	Elective termination of pregnancy due to fetal cleft lip and palate

FSHR = follicle-stimulating hormone receptor, sOHSS = spontaneous ovarian hyperstimulation syndrome.

In Case 1, the patient experienced a significant delay in diagnosis due to a missed detection, which postponed critical treatment and accelerated disease progression. This highlights a severe dereliction of duty in timely clinical evaluation. In Case 4, misdiagnosis led to an unnecessary surgical procedure, compounding the patient’s physical and psychological distress-particularly amid the emotional challenge of coping with fetal abnormalities. The inefficiency not only intensified the patient’s suffering but also imposed additional financial burdens. These cases underscore the urgent need for accountability and systematic improvement in diagnostic processes.

## 4. Conclusion

This case series describes 4 pregnant women with sOHSS and severe primary hypothyroidism, highlighting the critical importance of thyroid function screening in the differential diagnosis of adnexal masses during natural pregnancy. Clinicians should consider sOHSS when encountering multicystic ovarian enlargement, particularly in patients with underlying thyroid or other endocrine disorders. Early diagnosis and medical management, including LT4 therapy, can lead to the resolution of ovarian cysts and prevent disease progressing and unnecessary surgical procedures. Unnecessary oophorectomy carries significant risks, including surgical complications, premature menopause, cardiovascular disease, osteoporosis, and psychological sequelae. Surgical intervention should be reserved for complications such as torsion, rupture, or necrosis, with every effort made to preserve ovarian tissue. The lack of a multidisciplinary team (MDT) discussion in these cases may have stemmed from a failure to recognize hypothyroidism as the primary etiology. Future management protocols should mandate MDT involvement in similar scenarios to optimize LT4 titration, avoid unnecessary interventions, and improve obstetric outcomes.

## Acknowledgments

The authors extend their sincere appreciation to the patients for their participation, which made this work possible, and to the professionals and researchers involved in the study.

## Author contributions

**Data curation:** Wenming Zhuang.

**Formal analysis:** Wenming Zhuang, Kaiheng Zhang.

**Investigation:** Lingfang Ye.

**Methodology:** Ruili Shao, Kaiheng Zhang.

**Project administration:** Wenming Zhuang.

**Validation:** Lian Wu.

**Writing – original draft:** Wenming Zhuang, Lian Wu.

**Writing – review & editing:** Wenming Zhuang.
